# Uterine Fibroid Torsion during Pregnancy: A Case of Laparotomic Myomectomy at 18 Weeks' Gestation with Systematic Review of the Literature

**DOI:** 10.1155/2017/4970802

**Published:** 2017-04-24

**Authors:** Annachiara Basso, Mariana Rita Catalano, Giuseppe Loverro, Serena Nocera, Edoardo Di Naro, Matteo Loverro, Mariateresa Natrella, Salvatore Andrea Mastrolia

**Affiliations:** ^1^Department of Obstetrics and Gynecology, Azienda Ospedaliera Universitaria Policlinico di Bari, School of Medicine, Università degli Studi di Bari “Aldo Moro”, Bari, Italy; ^2^School of Nursing, Azienda Ospedaliera Universitaria Policlinico di Bari, School of Medicine, Università degli Studi di Bari “Aldo Moro”, Bari, Italy

## Abstract

Uterine myomas are the most common benign growths affecting female reproductive system, occurring in 20–40% of women, whereas the incidence rate in pregnancy is estimated from 0.1 to 3.9%. The lower incidence in pregnancy is due to the association with infertility and low pregnancy rates and implantation rates after in vitro fertilization treatment. Uterine myomas, usually, are asymptomatic during pregnancy. However, occasionally, pedunculated fibroids torsion or other superimposed complications may cause acute abdominal pain. There are many controversies in performing myomectomy during cesarean section because of the risk of hemorrhage. Nevertheless, the majority of indication arises before labor and delivery due to acute symptoms leading to a discussion regarding the need for intervention during pregnancy. Therefore, we present a case of successful multiple laparotomic myomectomy at 17 + 2 weeks of gestational age and a systematic review of the literature in order to clarify the approach to this pathologic condition and its effect on pregnancy outcome.

## 1. Introduction

Uterine myomas are the most common benign growths affecting female reproductive system, occurring in 20–40% of women [1], whereas the incidence rate in pregnancy is estimated from 0.1 to 3.9%. The lower incidence in pregnancy is due to the association with infertility and low pregnancy rates and implantation rates after in vitro fertilization treatment [2]. Uterine myomas, usually, are asymptomatic during pregnancy. However, occasionally, pedunculated fibroids torsion or other superimposed complications may cause acute abdominal pain. Urinary and gastroenteric symptoms may occur due to the rapid increase in size in reason of hyperestrogenic environment and, consequently, compression and displacement of surrounding organs. Additionally, fibroids predispose to pregnancy complications, including early miscarriage, antepartum bleeding, preterm labor, premature rupture of membranes, fetal malpresentations, labor dystocia, and postpartum hemorrhage.

Conservative management with anti-inflammatory therapy is considered a gold standard, and surgery is generally avoided during pregnancy because of the risks of hysterectomy secondary to severe hemorrhage, pregnancy injury, and pregnancy loss [3]. The main conditions that induce inevitably the surgical procedure are the torsion of pedunculated fibroids or rare cases of necrosis, resultant inflammatory peritoneal reaction, and, finally, if symptoms persist after 72 hours of pharmacological therapy [4–7]. Therefore, the diagnosis needs a particular attention for the appropriate management choice. Surgical removal fibroids in pregnancy can be performed by laparotomy or laparoscopy technique taking into account the volume and location of nodules [1, 8].

Laparoscopy can be considered in selected cases such as small, subserous, pedunculated myomas.

There are many controversies in performing myomectomy during cesarean section because of the risk of hemorrhage [3]. Nevertheless, the majority of indication arises before labor and delivery due to acute symptoms leading to a discussion regarding the need for intervention during pregnancy.

Therefore, we present a case of successful multiple laparotomic myomectomy at 17 + 2 weeks of gestational age and a systematic review of the literature in order to clarify the approach to this pathologic condition and its effect on pregnancy outcome.

## 2. Case Report

Uterine myomas are usually asymptomatic during pregnancy. However, pedunculated fibroids torsion may occasionally cause acute abdominal pain [1].

Most cases of laparotomic myomectomy described in literature have been performed during a cesarean section due to the risk of managing them surgically at low gestational age [2–4]. We present a case of a successful multiple laparotomic myomectomy during the second trimester of pregnancy.

A 36-year-old, morbidly obese primigravida presented at our emergency room at 17 + 0 weeks of gestational age complaining of abdominal pain. At clinical examination, the uterus appeared to be of higher volume compared to the gestational age, the abdomen was painful but treatable, and the obstetrical examination was normal. The patient was then referred to US Unit of our Department for further evaluation. The sonographic assessment revealed the presence of three subserous uterine myomas located on anterior wall (maximum diameter: 13.2 cm), the right wall (maximum diameter: 12.6 cm), and the left wall (maximum diameter: 11.7 cm) of the uterus, respectively. All myomas were vacuolated inside as for suspected necrosis. The scan also showed other multiple myomas less than 3 cm in size. Vital signs were monitored (blood pressure 140/90 mmHg, maternal heart rate 124 bmp, SO2 94%, apyretic). Amniotic fluid was normal and fetal well-being was preserved. Thus, the patient was admitted to the High-Risk-Pregnancy Unit. When collecting the medical history, the first trimester ultrasound scan, performed at 11 weeks' gestation, revealed the presence of the same lesions with a size of 10.8 cm, 10.2 cm, and 6.14 cm, respectively.

Laboratory studies demonstrated rising inflammatory markers (C-reactive protein: 354 mg/L; WBC: 16.92 × 10^3^ *μ*L).

**Figure 1:**
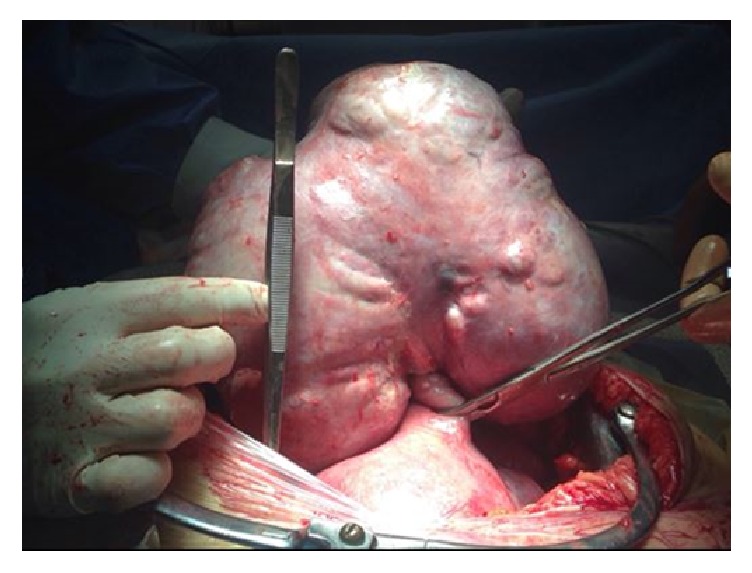
Myoma of the uterine fundus with evidence of torsion of its pedicle.

Due to the persistence of the symptoms, despite of two days of analgesic, antispastic, and antibiotic therapy, after multidisciplinary discussion, and a thorough counseling to inform the parents of the surgical and postoperative risks connected with uterine surgery during the gestation, the patient underwent surgery. Laparotomy approach by longitudinal skin incision, considering the volume and the position of the myomas, was performed under general anesthesia. Three huge bulky subserous pedunculated myomas were evidenced, the largest located at the uterine fundus, with a maximum diameter of 15 cm and a torsion of its pedicle (Figure 1). Furthermore, intra-abdominal adhesions were found within peritoneal cavity. Blunt dissection was undertaken to free the omentum and look for the appendix, which was normal. The three large myomas evidenced by ultrasound were removed and sent for pathologic examination. A pelvic drainage was left and removed 24 hours postoperatively. Pathology showed widespread phenomena of necrosis, especially in the myoma with torsion of its pedicle.

During the following nine days, the patient received antibiotics, low molecular heparin, and progesterone, and fetal heartbeat was checked daily. Considering the improvement in clinical condition, the patient was discharged with an indication to treatment with progesterone and low molecular heparin.

Three weeks later, at 21 weeks' gestation, the patient was admitted again due to abdominal pain. Obstetrical evaluation revealed cervical effacement and the transvaginal ultrasound scan showed a reduction of cervical length (18 mm), funneling, and sludge. An ultrasound scan was performed showing good fetal variables. Consequently, the therapy with progesterone was increased. The patient had a positive vaginal culture for* Staphylococcus haemolyticus*, urine culture was negative, and C-reactive protein resulted to be positive. Therefore, antibiotic therapy with macrolides was given, according to antibiogram result. A cervical cerclage was proposed to the patient, but she refused to undergo the procedure.

Hospitalization lasted for seven days; then the woman was discharged due to an improvement of her clinical condition. The patient underwent obstetric evaluation every two weeks until she presented in labor and delivered vaginally at 38 + 1 weeks' gestation a healthy female newborn of 2940 g, appropriate for gestational age according to national growth curves [9]. Apgar score was 9/10 at 1′ and 5′ respectively.

## 3. Data Source and Literature Search

To identify potentially eligible studies, we searched PubMed, Scopus, and Cochrane Library (all from inception to 16 March 2017). No language restrictions were initially applied. We used a combination of key words and text words represented by “myomectomy,” “myoma,” and “pregnancy.”

Two reviewers (Annachiara Basso and Mariana Rita Catalano) independently screened the titles and abstracts of records retrieved through database searches. Both reviewers recommended studies for the full-text review. The screen of full-text articles recommended by at least one reviewer was done independently by the same two reviewers and assessed for inclusion in the systematic review. Disagreements between reviewers were resolved by consensus. For all full-text manuscripts, reference lists were analyzed in order to find additional eligible studies.

## 4. Results

The electronic database search provided a total of 1855 results. After duplicate exclusion, there were 1611 citations left. Of these, 1508 were not relevant to the review based on title and abstract screening. 103 studies were considered for full-text assessment, of which 40 were excluded for the following reasons: we could not translate 31 articles, while nine papers could not be retrieved even after international librarian search.

**Figure 2:**
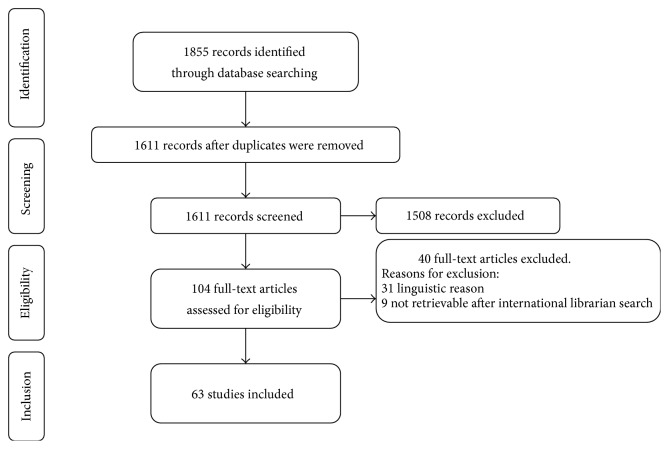
Study selection process.

Overall, 63 [3–6, 10–67] articles were incorporated for further assessment. The study selection process is shown in Figure 2. The main characteristics of the selected studies are included in Table 1.

## 5. Discussion

Our review included 197 women undergoing myomectomy during pregnancy. The procedure was successful in 184 women, while in the remaining 13 cases a miscarriage or fetal demise happened after the myomectomy.

In 14 cases, a laparoscopic approach was chosen; in one case there was a vaginal surgery, while all the other cases for which the surgical information was available underwent laparotomy. These data confirm that the most used surgical intervention for myomas during pregnancy is the laparotomy route.

Maternal outcomes were favorable after myomectomy, with only two episodes of hemoperitoneum [33, 67], one uterine abscess [39], and only one woman requiring perioperative blood transfusion [61].

Moreover, the analysis of all reports was limited by two factors: (1) the heterogeneity of diagnostic information as well as descriptive data connected to operation and pathology examination which did not allow clear categorization of the pathology preoperatively and postoperatively and (2) the large amount of missing or unreported data.

## 6. Conclusion

Myomectomy is a feasible procedure if performed during pregnancy. Candidates need to be chosen carefully among those with symptomatic myomas, since abdominal surgery during pregnancy can be associated with an increased risk for the development of the great obstetrical syndromes, especially preterm labor and delivery.

## Figures and Tables

**Table 1:** Characteristics of the relevant studies.

Reference	Number of patients	Gestational age at diagnosis (weeks)	Gestational age at myomectomy (weeks)	Type of surgery	Fibroid maximum volume (cm)	Mode of delivery	Gestational age at delivery	Neonatal outcome (Apgar, birthweight, pH)
De Carolis et al., 2001	18	nd	13	LPT	8	CS	39	8/8, 3150 g
		nd	23	LPT	40	CS	38	8/8, 2670 g
		nd	19	LPT	14	VD	36	8/9, 3080 g
		nd	17	LPT	21	CS	38	8/9, 3060 g
		nd	19	LPT	15	Fetal demise at 19 weeks		
		nd	20	LPT	6	VD	41	9/9, 2970 g
		nd	19	LPT	12	CS	39	7/9, 3180 g
		nd	8	LPT	9	CS	40	9/9, 3300 g
		nd	12	LPT	8	CS	38	9/10, 2780 g
		nd	17	LPT	24	CS	38	9/9, 3900 g
		nd	15	LPT	10	CS	40	8/10, 3170 g
		nd	17	LPT	13	CS	39	9/10, 3100 g
		nd	6	LPT	15	nd	nd	nd
		nd	20	LPT	8	CS	39	9/10, 2860 g
		nd	10	LPT	16	CS	40	9/10, 3500 g
		nd	16	LPT	10	CS	39	9/10, 3930 g
		nd	13	LPT	14	CS	39	9/9, 3180 g
		nd	7	LPT	15	CS	38	9/10 - 2550 g

Domenici et al., 2014	1	16	16	LPT	20	CS	38	8/9 - 3250 g

Michalas et al., 1995	1	14	15	LPT	20	CS	39	2800 g

Danzer et al., 2001	1	12	12	LPT	10	CS	37	9/10, 3235 g;
								9/10, 2810 g

Lozza et al., 2011	1	12	16	LPT	18	CS	36	9/9, 2280 g

Joó et al., 2001	1	8	25	LPT	12	CS	40	3600 g

Çelik et al., 2002	5	nd	22	LPT	13	CS	38.6 +/− 1.1	10, 3200 g
		nd	18	LPT	10	CS	38.6 +/− 1.1	9, 3400 g
		nd	20	LPT	12	CS	38.6 +/− 1.1	10, 3600 g
		nd	16	LPT	15	CS	38.6 +/− 1.1	8, 3100 g
		nd	13	LPT	20	CS	38.6 +/− 1.1	9, 2800 g

Hasbargen et al., 2002	1	18	18	LPT	15	CS	36	8/9, 2495 g

Umezurike and Feyi-Waboso, 2005	1	19	19	LPT	32	VD	38	8/10, 3500 g

Usifo et al., 2007	1	13	13	LPT	17	CS	38	3990 g

Suwandinata et al., 2009	1	nd	18	LPT	nd	CS	37	8/9, 2950 g

Bhatla et al., 2009	1	8	19	LPT	28	VD	38	2740 g

Leite et al., 2009	1	1st trimester	17	LPT	10	CS	39	9/10, 3315 g

Isabu et al., 2010	1	14	14	LPT	nd	CS	37	2700 g

Leach et al., 2011	1	11	11	LPT	14	CS	40	9/9, 4356 g

Doerga-Bachasingh et al., 2012	1	9	10	LPT	15	CS	37	nd

Jhalta et al., 2016	1	13	13	LPT	16	VD	39	8/10, 3000 g

Kosmidis et al., 2015	1	10	10	LPS	8	nd	nd	nd

Saccardi et al., 2015	1	9	15	LPS	24	CS	41	4460 g, 7.2

Obara et al., 2014	1	6	13	VAG	6	VD	40	2775 g

Currie et al., 2013	1	11	11	LPS	8	nd	nd	nd

Kobayashi et al., 2013	1	21	21	LPT	8	CS	37	2730 g

MacCiò et al., 2012	3	8	19	LPS	11	CS	39	3150 g
		20	20	LPS	10	VD	40	3310 g
		20	20	LPS	nd	CS	39	3050 g

Shafiee et al., 2012	1	15	21	LPS	15	CS	38	nd

Ardovino et al., 2011	1	14	14	LPS	6	VD	40	3216 g

Müller Vranjes et al.	1	14	18	LPT	35	CS	33	10/10, 1750 g, 7.28

Son et al., 2011	1	18	18	LPS	9	VD	39	3740 g

Kasum 2010	1	15	15	LPT	9	VD	38	nd

Fanfani et al., 2010	1	25	25	LPS	9	VD	40	2950 g

Adeyemi et al., 2007	1	19	19	LPT	30	VD	39	7/10, 3500 g

Okonkwo and Udigwe, 2007	1	19	24	LPT	nd	CS	nd	nd

Dracea and Codreanu, 2006	1	12	13	LPT	24	VD	nd	nd

Melgrati et al., 2005	1	24	24	LPS	7	VD	39	9/9

Sentilhes et al., 2003	1	17	17	LPS	5	CS	37	3530 g

Lolis et al., 2003	13	nd	16	LPT	nd	CS	37	3340 g
		nd	15	LPT	nd	CS	39	3600 g
		nd	19	LPT	nd	CS	37	2970 g
		nd	16	LPT	nd	CS	36	3000 g
		nd	15	LPT	nd	Fetal demise at 15 weeks		
		nd	15	LPT	nd	CS	37	2740 g
		nd	16	LPT	nd	CS	38	3180 g
		nd	16	LPT	nd	CS	39	3515 g
		nd	16	LPT	nd	CS	39	3190 g
		nd	19	LPT	nd	CS	38	2920 g
		nd	17	LPT	nd	CS	38	3520 g
		nd	16	LPT	nd	CS	38	3000 g
		nd	15	LPT	nd	CS	29	1606 g

Donnez et al., 2002	1	Before pregnancy	25	LPT	22	CS	35	2280 g

Williamson, 1908	1	22	22	LPT	32	VD	23	Neonatal death

Stewart, 1906	1	20	20	LPT	24	VD	40	nd

Wittich et al., 2000	1	12	15	LPT	20	CS	37	9/9, 3275 g

Majid et al., 1997	1	17	18	LPT	24	Fetal demise 19 weeks		

Algara et al., 2015	1	18	18	LPS	7	VD	24	nd

Lockyer, 1914	1	21	21	LPT	nd	VD	40	2300 g

von Hoffmann, 1911	3	16	16	LPT	nd	VD	40	3630 g
		22	25	LPT	nd	Fetal demise at 25 weeks		
		14	15	LPT	nd	VD	40	nd

Andrews, 1910	1	Before pregnancy	9	LPT	nd	VD	40	nd

Swayne, 1908	2	20	20	LPT	nd	nd	nd	nd
		16	16	LPT	nd	VD	24	nd

Doran, 1906	1	20	21	LPT	10	VD	40	nd

Evans, 1899	1	20	20	LPT	7	nd	nd	nd

Exacoustòs and Rosati, 1993	13	nd	<26	nd	nd	N.G	40 (8), preterm > 32 (5)	nd

Burton et al., 1989	8	nd	13	LPT	18	VD	40	nd
		nd	15	LPT	14	Fetal demise 15 weeks		
		nd	nd	LPT	5	VD	40	nd
		nd	nd	LPT	5	VD	40	nd
		nd	nd	LPT	5	VD	40	nd
		nd	nd	LPT	5	VD	40	nd
		nd	nd	LPT	5	VD	40	nd
		nd	nd	LPT	5	nd	nd	nd

Rella et al., 1980	1	10	12	LPT	nd	VD	27	Neonatal death

Pelosi et al., 1995	1	13	15	LPS	6	CS	39	nd

Pelissier-Komorek et al., 2012	1	10	13	LPT	22	VD	35	2280 g

Mollica et al., 1996	18	8–17	10–19	LPT	>10	CS (17), VD (1)	nd	>7 (18), >2500 g (17), <2500 g (1)

Febo et al., 1997	3	nd	12–19	LPT	N.G.	CS (2), abortion (1)	37-38	nd

Bonito et al., 2007	5	nd	9–15	LPT	3.5–14.5	CS (2), VD (3)	38.2	9 +/− 0.83, 3200–4072 g

Vázquez Camacho et al., 2009	1	7	16	LPT	6.2	VD	40	9/9

Makar et al., 1989	1	12	17	LPT	13,500 g	CS	38	9/9, 3950 g

Horno Liria, 1962	1	16	16	LPT	nd	VD	40	3600 g

Alanis et al., 2008	1	7	12	LPT	30	VD	38	2330 g

Ardizzone, 1955	27	8	8	LPT	nd	nd	nd	nd
		8	8	LPT	nd	nd	nd	nd
		8	8	LPT	nd	Miscarriage at 9 weeks		
		24	24	LPT	nd	Fetal demise at 25 weeks		
		8	8	LPT	nd	Miscarriage at 8 weeks		
		16	16	LPT	nd	nd	nd	nd
		8	8	LPT	nd	nd	nd	nd
		8	8	LPT	nd	nd	nd	nd
		12	12	LPT	nd	Fetal demise at 14 weeks		
		20	20	LPT	nd	nd	nd	nd
		16	16	LPT	nd	nd	nd	nd
		20	20	LPT	nd	nd	nd	nd
		20	20	LPT	nd	nd	nd	nd
		12	12	LPT	nd	nd	nd	nd
		12	12	LPT	nd	Fetal demise at 13 weeks		
		8	8	LPT	nd	nd	nd	nd
		8	8	LPT	nd	nd	nd	nd
		12	12	LPT	nd	Fetal demise at 13 weeks		
		12	12	LPT	nd	nd	nd	nd
		16	16	LPT	nd	Fetal demise at 17 weeks		
		8	8	LPT	nd	nd	nd	nd
		12	12	LPT	nd	nd	nd	nd
		12	12	LPT	nd	nd	nd	nd
		12	12	LPT	nd	nd	nd	nd
		8	8	LPT	nd	nd	nd	nd
		12	12	LPT	nd	Fetal demise at 12 weeks		
		12	12	LPT	nd	nd	nd	nd

Cozzi, 1967	16	nd	12	LPT	nd	VD	40	nd
		nd	12	LPT	nd	VD	40	nd
		nd	8	LPT	nd	VD	40	nd
		nd	8	LPT	nd	VD	40	nd
		nd	16	LPT	nd	VD	38	nd
		nd	8	LPT	nd	VD	40	nd
		nd	12	LPT	nd	VD	38	nd
		nd	8	LPT	nd	VD	40	nd
		nd	16	LPT	nd	VD	40	nd
		nd	20	LPT	nd	VD	36	nd
		nd	8	LPT	nd	VD	40	nd
		nd	12	LPT	nd	VD	40	nd
		nd	12	LPT	nd	VD	40	nd
		nd	12	LPT	nd	VD	40	nd
		nd	16	LPT	nd	VD	40	nd
		nd	8	LPT	nd	VD	40	nd

Rochet et al., 1964	14	nd	nd	LPT	10	nd	nd	nd

Sciannameo et al., 1996	1	20	20	LPT	4	nd	nd	nd

nd, not determined; CS, cesarean section; VD, vaginal delivery; LPT, laparotomy; LPS, laparoscopy; VAG, vaginal surgery.
